# Fabrication and Characterization of Chitosan/Cellulose Nanocrystal/Glycerol Bio-Composite Films

**DOI:** 10.3390/polym13071096

**Published:** 2021-03-30

**Authors:** Kusmono, Muhammad Waziz Wildan, Fadhlan Ihsan Lubis

**Affiliations:** Department of Mechanical and Industrial Engineering, Faculty of Engineering, Universitas Gadjah Mada, Jln. Grafika No. 2, Yogyakarta 55281, Indonesia; m_wildan@ugm.ac.id (M.W.W.); fadhlan.ihsan.l@mail.ugm.ac.id (F.I.L.)

**Keywords:** chitosan, cellulose nanocrystal, bio-composite films, mechanical properties

## Abstract

Cellulose nanocrystal (CNC)-reinforced bio-composite films containing glycerol were produced using the solution casting technique. The influences of the addition of CNC (2, 4, and 8 wt%) and glycerol (10, 20, and 30 wt%) on the properties of the bio-composite films were studied in the present work. The resulting films were characterized by X-ray diffraction (XRD), Fourier transform infrared (FT-IR) spectroscopy, and thermogravimetry analysis (TGA), and according to their tensile, water absorption, and light transmission behavior. The introduction of 4 wt% CNC into the chitosan film did not affect the thermal stability, but the presence of 20 wt% glycerol reduced the thermal stability. The addition of 4 wt% CNC to the chitosan film increased its tensile strength, tensile modulus, and elongation at break by 206%, 138%, and 277%, respectively. However, adding more than 8 wt% CNC resulted in a drastic reduction in the strength and ductility of the chitosan film. The highest strength and stiffness of the chitosan bio-composite film were attained with 4 wt% CNC and 20 wt% glycerol. The water absorption and light transmission of the chitosan film were reduced dramatically by the presence of both CNC and glycerol.

## 1. Introduction

Currently, most food packaging films are made of synthetic polymers derived from fossil fuels. This is ascribed to their superior mechanical and barrier properties, easy processing, and low cost [1]. However, the application of synthetic polymers leads to environmental problems, owing to them not being easily degraded after use [2]. Therefore, many researchers have recently developed bio-based packaging films as a replacement for petroleum-based packaging films. Some of the main advantages of bio-based packaging films are their environmentally friendly nature, their biodegradability, and the nutritional value of their food products, as well as their contribution to the maintenance of food quality, and provision of microbial safety to users as a protective barrier [3,4]. Due to their environmentally friendly and biodegradable properties, many natural biopolymer materials have been widely used to make biodegradable food packaging materials, including chitosan, cellulose, hemicellulose, lignin, pectin, starch, agar, *Eucommia ulmoides* gum, and natural rubbers [1,5,6,7].

Chitosan is a natural linear polysaccharide containing 1,4-linked 2-amino-deoxy-β-d-glucan, which is part of the deacetylated derivative of chitin [8]. Chitosan is widely demonstrated to be a good candidate for biodegradable food packaging films because of its non-toxicity, environmental-friendliness, bio-functionality, biocompatibility, and strongly antimicrobial and antibacterial activities [8]. However, chitosan films have some disadvantages including their solubility in semi-aquatic environments and propensity to dissolve in acid solution, low moisture resistance, and poor mechanical properties. To overcome the main drawbacks of chitosan, several suggestions have been made to improve its properties such as by the incorporation of nanofillers, plasticizers, and/or cross-linking agents. Numerous studies on chitosan films have been investigated by previous researchers using different nanofillers such as cellulose nanocrystal [8,9], nano clay [10], starch palm cellulose nanocrystal (CNC) [11], and carbon nanotubes [12].

Among the various nanofillers, CNC has attracted significant interest as a potential nano-reinforcement for chitosan films because of its sustainability, abundance, large surface area to volume ratio, lightness, and high mechanical strength [13]. CNC is defined as a nanometer-sized crystal rod-shaped particle produced as a stable aqueous colloidal suspension, and is typically isolated from a cellulose source by acid hydrolysis [13]. Many studies on chitosan/CNC bio-composite films have been reported by previous researchers. It has been reported that the incorporation of 3–5 wt% CNC to chitosan increases the tensile strength, tensile modulus (significantly so), and barrier characteristics owing to the percolating network formation and strong interaction between CNC and the chitosan matrix [8,9]. It has also been found that the thermal resistance of chitosan is unchanged by the presence of CNC whereas the hydrophilicity of chitosan increases, as indicated by the decreased contact angle value. Moreover, the crystallinity of chitosan films is enhanced with CNC addition. Corsello et al. [14] demonstrated that the tensile strength and modulus of chitosan film enhances with CNC, but decreases with both water contact angle and water vapor permeability.

Although numerous studies on chitosan/CNC bio-composite films have been reported [8,9,14,15,16,17,18], there are limited studies on the effects after adding both CNC isolated from ramie fibers and glycerol on the thermal resistance, tensile, water resistance, and light transmission behaviors. In the present work, chitosan/CNC bio-composite films containing different CNC contents (2, 4, and 8 wt%), without and with glycerol (10, 20, and 30 wt%), were produced using the solution casting technique. The CNC used in this study was produced from ramie fibers using sulfuric acid hydrolysis [19]. In this work, the effects on the thermal resistance, tensile, water resistance, and light transmission behaviors of chitosan film following addition of CNC and glycerol were determined. The structure of the films was analyzed by X-ray diffraction (XRD) and Fourier transform infrared (FT-IR), whereas the tensile properties of the films were measured using the tensile test.

## 2. Materials and Methods

### 2.1. Materials

Chitosan (high molecular weight chitosan, with a viscosity of 800–2000 cP and degree of deacetylation above 75%), glacial acetic acid, and glycerol were supplied by Sigma Aldrich, Singapore. The CNC used in the present study was prepared from the isolation of ramie fibers via sulfuric acid hydrolysis following the procedure described in our previous work [19].

### 2.2. Isolation of CNC

CNC was extracted from the chemically purified cellulose (CPC) of ramie fibers through sulfuric acid hydrolysis based on our previously published procedures [19]. Briefly, ramie fibers were first purified through chemical pre-treatments, including de-waxing, bleaching, and alkalization, to remove amorphous components. The obtained CPC was isolated via 58 wt% sulfuric acid hydrolysis at 45 °C for 30 min with a CPC/acid ratio of 1:20 under magnetic stirring. Then, to stop the reaction, the suspension was cooled with the addition of cold distilled water (approximately 5 °C) at a suspension/cold water ratio of 1:20 (*v*/*v*). The CNC suspension was centrifuged at 4000 rpm for 15 min to remove the acid solution. CNC precipitates were collected and rinsed with distilled water until rinses were neutral. The ultra-sonication of CNC suspension was then ultrasonicated for 1 min with 50% amplitude to obtain a uniform CNC suspension. The obtained CNC had rod-shaped particles with high crystallinity (90.77%), an average diameter of 6.67 nm, and an average length of 145.61 nm, consistent with our previous studies [19].

### 2.3. Preparation of Chitosan/CNC/Glycerol Bio-Composite Films

The films were produced via a solution casting method, as previously described, with a few modifications [8,20]. The chitosan solution (2%, *w*/*v*) was produced by dissolving chitosan powder in (1%, *v*/*v*) aqueous acetic acid solution using a mechanical stirrer at 90 °C for 15 h at a constant speed of 300 rpm, and subsequently cooled to room temperature. The chitosan solution was mixed with CNC of varying amounts (0, 2, 4, and 8 wt%) using a mechanical stirrer at 70 °C for 2 h with a speed of 300 rpm. This solution was then poured into a 200 mm × 200 mm acrylic mold and cooled to room temperature. The films containing 0, 2, 4, and 8 wt% CNC are referred to as CS, CS/CNC2, CS/CNC4, and CS/CNC8, respectively. Additionally, the chitosan/CNC/glycerol bio-composite films were also prepared by adding different amounts of glycerol (10, 20, and 30 wt%) on solid CS into the chitosan/CNC solution containing 4 wt% CNC, and stirred using a mechanical stirrer at 70 °C for 2 h at a speed of 300 rpm. Subsequently, the solution was poured into the acrylic mold and then cooled to room temperature for producing the films. Furthermore, the bio-composite films with 4 wt% CNC consisting of 10, 20, and 30 wt% glycerol are referred to as CS/CNC4/G10, CS/CNC4/G20, and CS/CNC4/G30, respectively.

### 2.4. X-ray Diffraction (XRD) Analysis

The X-ray diffraction of the films was analyzed using an X-ray diffractometer (PHILIPS, Malvern, United Kingdom) fixed with CuKα radiation (λ = 0.1541 nm) in the 2θ range of 5−30° with a step increment of 2θ = 0.02° at 40 kV and 30 mA using the 2θ mode for scanning.

### 2.5. Fourier Transform Infrared (FT-IR) Spectra Analysis

The FT-IR analysis of the films was carried out by recording spectra with an FT-IR spectrophotometer (IRPrestige21 machine from Shimadzu Corporation, Tokyo, Japan) in the wavenumber range of 4000–400 cm^−1^ at a resolution of 4 cm^−1^.

### 2.6. Thermal Stability

The thermal stability of the films was determined by thermogravimetry analysis (TGA) (STA7200 HITACHI, Hitachi High-Technologies Corporation, Tokyo, Japan) at a temperature range of 30–600 °C with a heating rate of 10 °C/min under nitrogen conditions.

### 2.7. Tensile Properties

The tensile properties of the films were measured through a tensile test. The tensile test was conducted by a universal testing machine (HT-2402, HUNG TA Instrument Co. Ltd., Taichung City, Taiwan) at a crosshead speed of 5 mm/min following the ASTM D638 Type IV standard. The tensile strength, tensile modulus, and elongation at break were determined through the tensile test at room temperature. The films were stretched with an initial grip separation of 33 mm, and four samples were tested for each measurement.

### 2.8. Water Absorption

Water absorption was measured using the following procedure. Films with dimensions of 12 mm × 12 mm × 0.09 mm were placed in a desiccator for 24 h, followed by weighing to measure the dry mass. The films were then immersed entirely in the distilled water in a sealed beaker. The weight of the films was measured followed periodic immersion, and the water absorption ($W_{A}$) of the films was then determined according to Equation (1) below:

(1) $$W_{A}=\;\frac{\left(W_{i}-W_{o}\;\right)}{W_{o}}\;\times \;100\%$$

where $W_{i}$ and $W_{o}$ depict the immersed and dried weights of the films, respectively.

### 2.9. Light Transmittance Analysis

The light transmittance of the films was analyzed using an Ocean Optics UV–vis spectrometer (model USB4000 Fiber Optic Spectrometer) at room temperature and related to the film thicknesses according to the Beer-Lambert law. The light transmittance analysis was performed at a wavelength range of 300–800 nm with a 0.2 nm spectral bandwidth.

## 3. Results and Discussion

### 3.1. XRD Analysis

**Figure 1:**
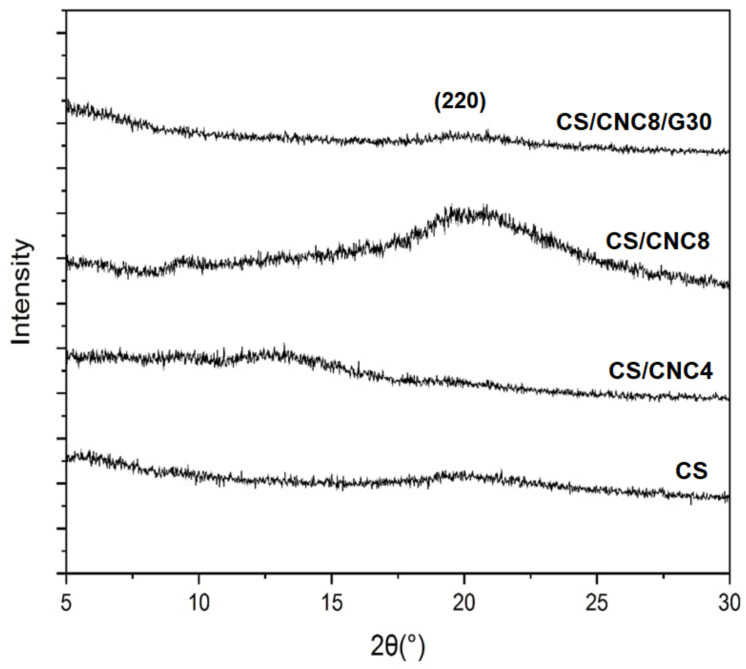
X-ray diffraction (XRD) patterns of chitosan and its bio-composite films.

The XRD patterns of the chitosan film (CS) and its bio-composite films consisting of either 4 (CS/CNC4) or 8 wt% CNC (CS/CNC8), and 8 wt%CNC/30 wt% glycerol (CS/CNC8/G30) are shown in Figure 1. A broad peak at around 2θ = 20° was observed in the diffractogram of the chitosan corresponding to the crystal plane of (220) of the crystalline structure [8]. A peak at around 2θ = 13° was exhibited in the diffractogram of the chitosan bio-composite consisting of 4 wt% CNC, indicating a hydrated crystalline structure [16,21]. Furthermore, the addition of 8 wt% CNC led to increased peak intensity at 2θ = 20°. This increase in peak intensity might be attributable to the transcrystallization effect [8]. The transcrystallization impact corresponds to the crystal orientation of the semicrystalline matrix perpendicular to the cellulose nanocrystalline [22]. Crystallization of the polymer matrix is enhanced by nanocrystalline cellulose, resulting in a transcrystalline layer around the CNC [23]. The effect of addition of 30 wt% glycerol on the chitosan/CNC bio-composite film structure is also shown in Figure 1. The presence of 30 wt% glycerol led to a drastic decrease in the intensity of the peak at 2θ = 20°. This suggests that the introduction of 30 wt% glycerol reduced the crystalline structure of the film. In other words, adding glycerol lowered the crystallinity of the film, influencing the mechanical properties of the films. Glycerol is a polyalcohol and is the most used cryosolvent because of its antifreeze properties. Glycerol might act as an antinucleation agent in the crystallization process, resulting in lower crystallinity of the film [24].

### 3.2. FT-IR Analysis

**Figure 2:**
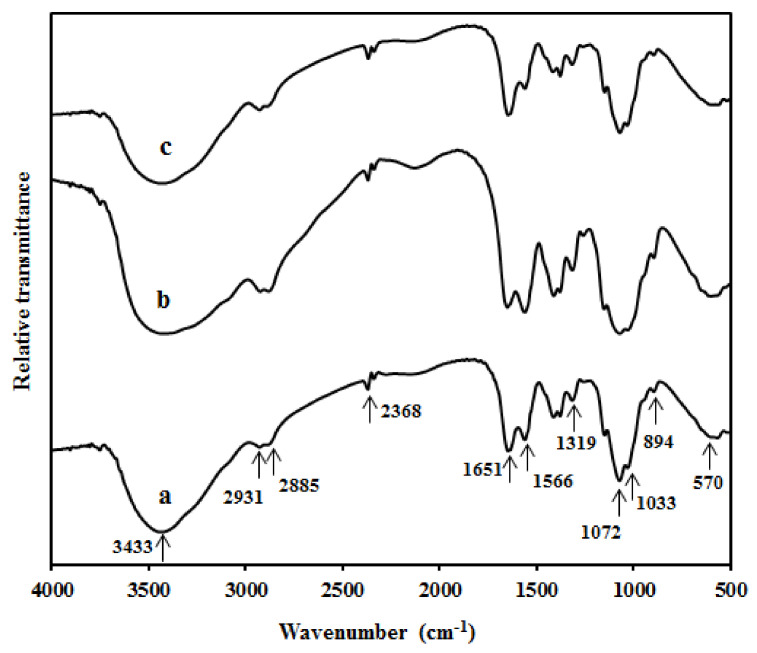
Fourier transform infrared (FT-IR) spectra of (**a**) chitosan, (**b**) bio-composite film with 4 wt% cellulose nanocrystal (CNC), and (**c**) bio-composite film with 4 wt% CNC and 20 wt% glycerol.

Figure 2 displays the FT-IR curves of the chitosan film and its bio-composite films with and without 20 wt% glycerol. All the samples displayed the same spectra, as indicated by peaks at 570, 1033, 1072, 1319, 1566, 1651, 2885, 2931, and 3433 cm^−1^ (Figure 2a). The peak at 570 cm^−1^ corresponds to the skeletal mode vibrations of the pyranose ring [19,25]. The characteristic peaks at 1033, 1072, and 1319 cm^−1^ reflect the stretching of C–O, bending of O–H, and bending of C–H, respectively [8,26]. The peaks at 1566 and 1651 cm^−1^ reflect the vibrational mode of the amide I and II groups, respectively [27]. The peaks at 2885 and 2931 cm^−1^ correspond to asymmetric and symmetric C–H vibrations, respectively. The sharp peak at 3433 cm^−1^ is associated with the O–H vibrations caused by intramolecular hydrogen bonding [28]. After incorporating 4 wt% CNC into the chitosan matrix (Figure 2b), the intensity of the peak at 3433 cm^−1^ increased, confirming the formation of hydrogen bonding between chitosan and CNC [29]. Moreover, the intensity of the peaks at 1566 and 1319 cm^−1^ slightly increased with the presence of CNC [8]. Furthermore, no new peak appeared in the spectrum of the chitosan/CNC4 film consisting of 20 wt% glycerol, indicating that the introduction of glycerol did not change the functional groups according to the film spectrum (Figure 2c).

### 3.3. Thermal Stability

**Figure 3:**
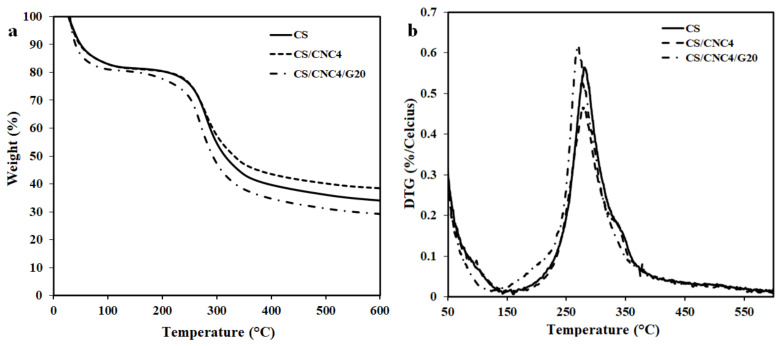
Thermogravimetry analysis (TGA) (**a**) and derivative thermogravimetry analysis (DTG) curves (**b**) of chitosan and its bio-composite films.

Figure 3a depicts the TGA curves of the chitosan (CS) and its bio-composite films consisting of 4 wt% CNC (CS/CNC4) and 20 wt% glycerol (CS/CNC4/G20). All the films exhibited similar behavior, with three main thermal degradation steps at temperature ranging from 27 to 600 °C. The first step (50–100 °C) is ascribed to the elimination of water molecules from films. Moreover, the weight loss may be related to the dehydration of loosely bound water and evaporation of low molecular weight compounds in the films [11]. The second stage occurred over a range of temperatures from 140 to 395 °C. In this stage, the weight loss is probably associated with the decomposition of glycerol, CNC, and chitosan. The main processes involved in CNC decomposition at the temperature range of 240–335 °C are cleavage of cellulose glycosidic bonds, rearrangement, dehydration, and breakdown reactions with C=O, water, and low-volatility molecular compounds [18,30]. Major decomposition of chitosan took place at approximately 140–310 °C through the depolymerization of the chitosan chains, including the deacetylation and cleavage of glycosidic linkages through dehydration and delamination [18,30]. The third step occurred in the temperature range of 395–600 °C. This is associated with the oxidation and breakdown of char into gas products of lower molecular weight [9].

Furthermore, the effect on thermal resistance of the addition of both 4 wt% CNC and 20 wt% glycerol into the chitosan film was investigated by determining the maximum degradation temperature (T_max_; the temperature at the maximum mass loss rate) based on the derivate thermogravimetry analysis (DTG) curves, as presented in Figure 3b. The T_max_ values of the films of chitosan, with 4 wt% CNC, and containing 20 wt% glycerol were 279, 280, and 270 °C, respectively. This indicates that the presence of 4 wt% CNC did not affect the thermal resistance, but the presence of 20 wt% glycerol reduced the T_max_ by 10 °C. In other words, the incorporation of 20 wt% glycerol decreased the thermal stability of the chitosan/CNC bio-composite films. This might be ascribed to the lower thermal degradation of glycerol, where glycerol degradation occurs at 160–200 °C [31]. In addition, a drop in the thermal stability of the films due to addition of glycerol is probably attributable to the evaporation of glycerol at a relatively low temperature range of 120–260 °C [32]. A similar finding has also been demonstrated in nanoclay-reinforced camelina gum-based films, where the addition of glycerol decreases their thermal stability [33]. The decreased thermal stability from the presence of glycerol has also been reported by previous researchers in bio-composite films based on camelina gum/nanoclay [31] and polyvinyl alcohol/bacterial cellulose [34].

### 3.4. Tensile Properties

**Figure 4:**
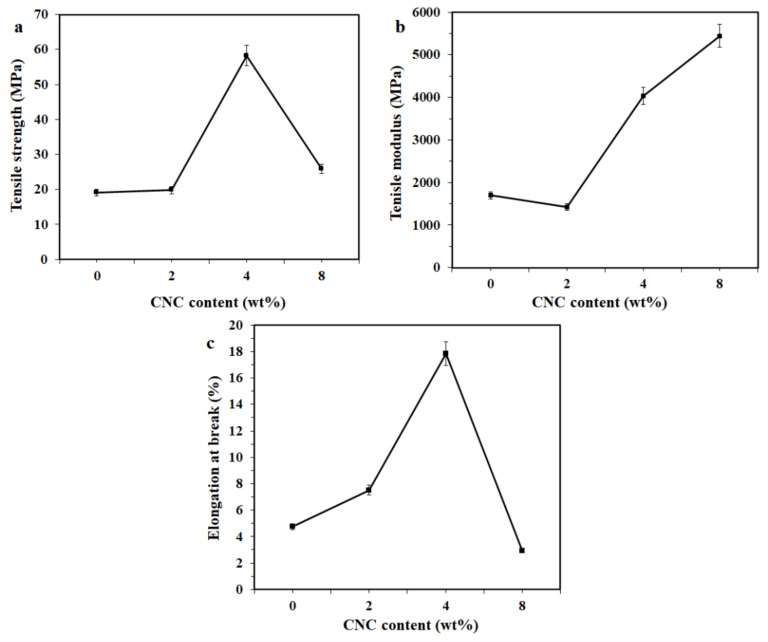
(**a**) Tensile strength of the chitosan/cellulose nanocrystal (CNC) bio-composite films with varying CNC contents, (**b**) tensile modulus, and (**c**) elongation at break.

Figure 4 presents the tensile properties of the chitosan/CNC bio-composite films with varying CNC contents. The influence of added CNC on the tensile strength of chitosan/CNC is demonstrated in Figure 4a. The tensile strength of the chitosan film was determined to be 19.06 MPa, while the tensile strength of the chitosan film containing 2 and 4 wt% CNC increased to 19.77 and 58.27 MPa, respectively. This indicates that the addition of CNC up to 4 wt% into the chitosan increased the tensile strength remarkably. The tensile strength of the chitosan film under study was one-fifth of that reported by Khan et al. [8]. This is probably attributable to the different molecular weight of the used chitosan matrix, where Khan et al. used a higher molecular weight than that of the chitosan studied in [8]. It is noteworthy that the tensile strength increased remarkably by 206% compared to that of the chitosan film. This is attributed to the strong interfacial adhesion between CNC and the chitosan matrix through the occurrence of hydrogen bonding, encouraging the electrostatic interaction of polyelectrolyte complexes between the cationic groups of chitosan and anionic sulfate groups of CNC [15,16,29]. An enhanced tensile strength of the chitosan film due to CNC has also been demonstrated by other researchers [8,18]. The highest tensile strength achieved in this study (58.07 MPa) with the addition of 4 wt% CNC was higher than that demonstrated by Xu et al. [18], but was still lower than that found by Khan et al. [8]. This difference might be ascribed to the differences in the molecular weight and the fiber source of the obtained CNC. Furthermore, the tensile strength drastically decreased because of the presence of 8 wt% CNC, but was still higher than that of the chitosan film. This is probably related to the weak interaction between CNC and the chitosan matrix, owing to the formation of CNC agglomerates, which acted as stress concentration points with poor dispersion between the –COO– and –NH^3+^ moieties resulting from charge neutralization [35,36]. Similar findings were also demonstrated by Xu et al., where the tensile strength of the chitosan/CNC film decreased as the CNC content exceeded 5 wt% [18]. The effect on the tensile modulus of chitosan film resulting from the addition of CNC is displayed in Figure 4b. The tensile modulus of the chitosan film was 1694 MPa. Adding 4 wt% CNC into the chitosan matrix remarkably increased the tensile modulus by 138%. At 8 wt% CNC loading, the tensile modulus was determined to be 5540 MPa, an improvement of 221% compared to the chitosan film. The increase in the tensile modulus at 4 and 8 wt% CNC might be associated with the presence of stiff CNC particles [8]. Furthermore, the influence of additional CNC on the elongation at break of the films is presented in Figure 4c. The elongation at break value was determined to be 4.74% for the chitosan film and 7.52%, 17.87%, and 2.92% for 2, 4, and 8 wt% CNC, respectively. This indicates that the incorporation of CNC up to 4 wt% increased the ductility, suggesting that the ductility can be increased by the presence of 2 and 4 wt% CNC. This increase in ductility for the films containing 2 and 4 wt% might be related to the better dispersion of CNC in the chitosan matrix, which does not guarantee the mobility of the chitosan chain, resulting in increased ductility. Moreover, the addition of 8 wt% CNC drastically reduced film ductility. This decrease was probably caused by the limited mobility of the chitosan chain with the addition of a high amount of CNC. A decrease in the ductility of chitosan films due to the incorporation of a high CNC content was also reported by Li et al. [27]. From Figure 4b,c, it can be seen that there was strong simultaneous enhancement of stiffness and ductility for the addition of 4 wt% CNC. This might be associated with the best dispersion of CNC in the chitosan matrix where CNC was dispersed uniformly in the nanometer scale in the chitosan matrix, leading to strong hydrogen bonding between the CNC and chitosan molecular chains, resulting in the formation of a rigid network of CNC, which causes an increase in the tensile modulus and elongation at break of the films [37]. Similar findings were also reported by Sun et al. [6] where the maximum values in tensile strength, tensile modulus, and elongation at break were achieved for *Eucommia ulmoides* gum film containing 4 wt% CNC. However, the tensile strength, tensile modulus, and elongation at break decreased drastically as the CNC content was further increased to 8 wt%. Furthermore, in terms of tensile strength, the obtained chitosan bio-composite film containing 4 wt% CNC exhibited a higher tensile strength (58 MPa) compared to other commercial food packaging films such as low-density polyethylene (8–31 MPa), liner low-density polyethylene (20–45 MPa), high-density polyethylene (17–45 MPa), and polypropylene (31–43 MPa) [38]. However, the resulting bio-composite films based on chitosan and CNC still had a much lower elongation at break (10%) than the other commercial films (100–1200%) [38].

**Figure 5:**
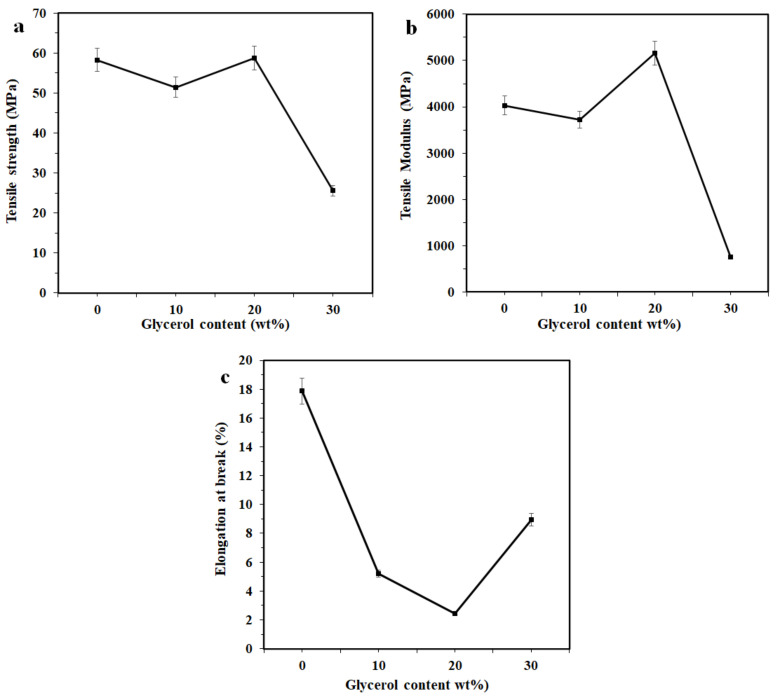
(**a**) Tensile strength of the chitosan/CNC/glycerol bio-composite films with varying glycerol contents, (**b**) tensile modulus, and (**c**) elongation at break.

Figure 5 displays the tensile properties of the bio-composite film with 4 wt% CNC as a function of glycerol content. The tensile strength of the bio-composite films with 10, 20, and 30 wt% glycerol was found to be 52, 59, and 26 MPa, respectively (Figure 5a). This indicates that the tensile strength of the film declined slightly with the presence of 10 wt% glycerol while increased slightly at 20 wt% and then decreased drastically at 30 wt% glycerol. A slight increase in tensile strength at 20 wt% glycerol might be associated with a better dispersion of CNC in the polymer matrix. At 30 wt%, a drastic decrement in tensile strength was associated with more dominant plasticizing effect of glycerol. Moreover, as a plasticizer, glycerol is a small oligomer that can penetrate macromolecules, which increases the free volume available between polymer chains, leading to a reduction in the physical entanglements and finally resulting in decreased tensile strength [17].

Figure 5b presents the influence of adding glycerol on the tensile modulus of chitosan/CNC/glycerol bio-composite films. The tensile modulus values were found to be 3722 MPa for 10 wt%, 5160 MPa for 20 wt%, and 750 MPa for 30 wt% glycerol. This suggests that the presence of 10 and 30 wt% glycerol decreased the tensile modulus, but the presence of 20 wt% increased. This trend is similar to that of the tensile strength, as previously discussed. The reduction in the tensile modulus for 10 and 30 wt% glycerol can probably be ascribed to the plasticizing effect of glycerol and its effect was more dominant at 30 wt. Moreover, the introduction of 20 wt% glycerol into the chitosan/CNC bio-composite films enhanced their tensile modulus. This enhancement is probably attributable to greater domination of the stronger hydrogen bonding of the chitosan-chitosan intermolecular interaction compared with the chitosan-glycerol [39]. A drastic drop in the tensile strength and tensile modulus of the bio-composite films containing 30 wt% CNC is attributed to the weak intramolecular interaction between the chitosan chains, supported by the formation of hydrogen bonds between chitosan molecules and glycerol, producing the lowered tensile strength and tensile modulus [39]. Similar findings were also reported by Qi et al. [33], who found that camelina gum/nanoclay/glycerol films with low glycerol concentration (15 wt%) show higher tensile strength and modulus compared to films without glycerol. However, the presence of a high glycerol content (30 and 45 wt%) decreases both the tensile strength and modulus due to a weak intramolecular attraction between the chains in polysaccharides and promotes the formation of hydrogen bonding between polysaccharides and glycerol, resulting in a decrease in the strength and stiffness of the films. The elongation at break as a function of the glycerol content of the chitosan/CNC bio-composites is shown in Figure 5c. It was found that the incorporation of 10–20 wt% glycerol led to drastically reduced ductility, but increased the ductility at 30 wt%. These results unexcepted as the ductility of the films should increase due to the presence of glycerol. This observation is attributed to CNC having a more dominant reinforcing effect than glycerol. It could be deduced that adding glycerol lowered the tensile characteristics of the chitosan/CNC bio-composite films.

### 3.5. Water Absorption

**Figure 6:**
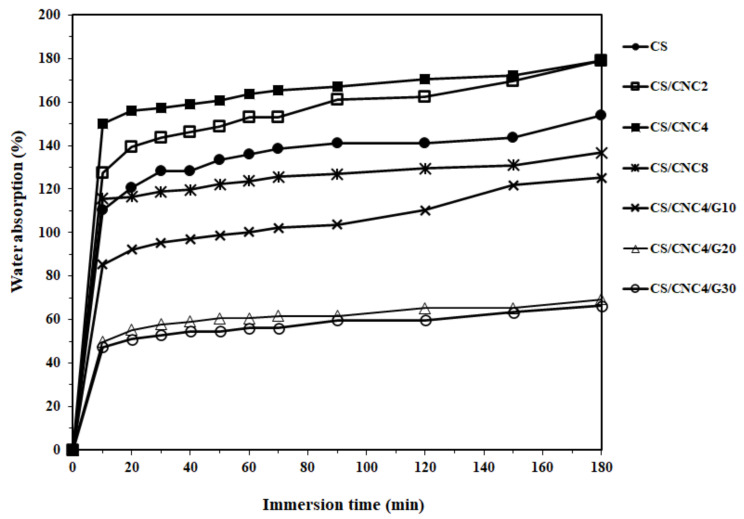
Water absorption curves of chitosan and its bio-composite films at various CNC and glycerol contents.

Figure 6 demonstrates the percentage of water absorption of the chitosan and its bio-composite films at various CNC and glycerol contents. All the samples exhibited an increase in water absorption with increasing immersion time. In the initial stage, a rapid linear increase in the water absorption was observed, which then slowed down and reached a plateau. This suggests that the water absorption characteristic of bio-composite films followed Fick’s law.

After an immersion time of 180 min, the water absorption of all chitosan/CNC bio-composite films containing 0, 2, 4, and 8 wt% CNC increased to 153.8, 179.1, 179, and 136.7%, respectively. This suggests that the water resistance decreased for chitosan films with 2 and 4 wt% CNC, but increased for the film containing 8 wt% CNC. The increased water absorption of the films containing 2 and 4 wt% might be attributable to both CNC and chitosan being hydrophilic materials, where hydrogen bonds easily form in water, thereby increasing water absorption [18]. The increased water absorption of the film containing 4 wt% CNC is consistent with the FT-IR curve, showing a sharper intensity of the hydrophilic functional group band at 3500–3200 cm^−1^ (reflecting O–H stretching). Furthermore, an increase in the water resistance of the film with 8 wt% CNC related to the chitosan film was probably caused by a barrier effect attributed to large CNC agglomerates that physically impeded the infiltration of water molecules [14].

The water absorption of the chitosan/CNC bio-composite films as a function of glycerol content is also shown in Figure 6. The water absorptions of the chitosan/CNC films containing different glycerol contents of 10, 20, and 30 wt% were 125%, 69%, and 66%, respectively. The water uptake drastically reduced from 179% to 125%, 69%, and 66% in the chitosan/CNC film plasticized with 10, 20, and 30 wt% glycerol, respectively, compared to the unplasticized film. This indicates that there was a drastic reduction in the water absorption of the bio-composite films containing glycerol. Therefore, the addition of glycerol improved the water resistance of the chitosan/CNC bio-composite films. The reason why the presence of glycerol increased the water resistance of the chitosan/CNC bio-composite films is attributed to the chemical structure of glycerol. As a result of its alkane backbone being surrounded by three O–H groups, hydrogen bonds are preferentially formed with glycerol rather than through the NH groups present in chitosan due to the difference in electronegativity [40]. Furthermore, the hydroxyl groups in glycerol may impede the occurrence of hydrogen bonds between chitosan and water molecules owing to the establishment of a complex network of strong hydrogen bonds between the chitosan, CNC, and glycerol. Moreover, the decreased water absorption of the bio-composite film consisting of glycerol is ascribed to the increased intermolecular hydrogen bonding between the chitosan matrix, cellulose nanocrystal, and glycerol, producing a decrease in the number of free O–H groups and lowering the diffusion of the water molecules. The reduction in water absorption of the bio-composite films with the presence of glycerol was also supported by the FT-IR spectrum, which exhibited a low intensity of the hydrophilic functional group band at 3500–3200 cm^−1^ (representing O–H stretching) [41]. Other researchers have also observed similar findings, i.e., that the water absorption of chitosan bio-composite films decreases in the presence of glycerol [10,40,41,42,43,44].

### 3.6. Light Transmittance Analysis

**Figure 7:**
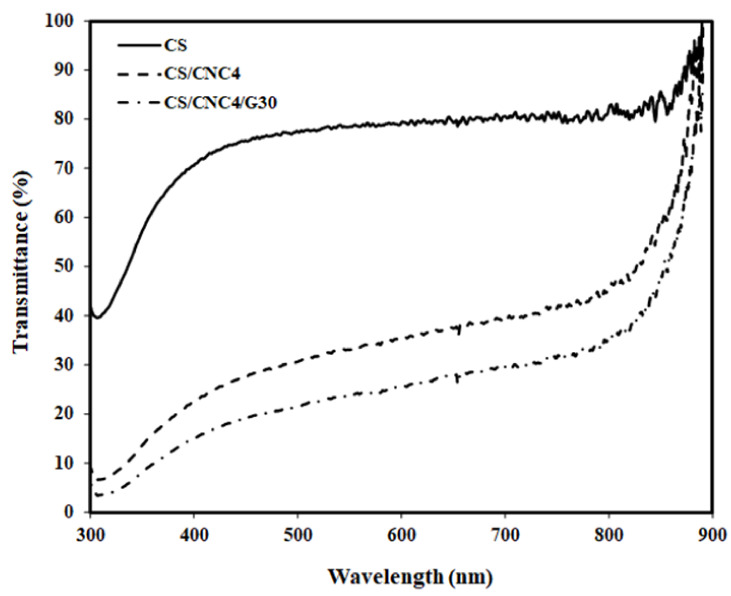
Light transmittance of the chitosan films and its bio-composite with 4 wt% CNC and with 30 wt% glycerol.

The light transmittance of the chitosan film (CS) and its bio-composites with 4 wt% CNC (CS/CNC4) and with 30 wt% glycerol (CS/CNC4/G30) is presented in Figure 7. At all analyzed wavelength ranges, both bio-composite films consisting of 4 wt% CNC and 30 wt% glycerol exhibited much lower light transmittance than the chitosan. The light transmittance decreased in the following order: CS > CS/CNC4 > CS/CNC4/G30. This indicates that adding CNC drastically decreased the light transmittance of the chitosan. This suggests that the transparency of the chitosan decreased remarkably due to the presence of CNC. This is attributed to the light path’s blockage through the polymer matrix resulting from the strong interface interaction between the CNC and the chitosan matrix [40,45]. Moreover, this decrease in transparency was also due to the increase in light scattering by the cellulose nanocrystals [46,47]. According to Tibolla et al. [45], a decrease in the light transmission of composite films can be influenced by the size and dispersion of nanofillers in the polymer matrix [42]. The decreased transmittance of the bio-composite films because of the presence of CNC was also reported by previous researchers using different biopolymer matrices such as agar [5], chitosan [18], and alginate [48]. The decreased light transmittance of the chitosan film resulting from the presence of nanoclay was also demonstrated in a previous study [33]. Furthermore, the chitosan/CNC bio-composite films plasticized with 30 wt% glycerol exhibited lower light transmittance than unplasticized films. This suggests that adding glycerol decreased the transparency of the chitosan/CNC bio-composite films. This is probably associated with the existence of a suitable contact area between the chitosan matrix, CNC, and glycerol [40]. Moreover, the film’s light transmittance was strongly influenced by the film thickness, in which a film with a higher thickness had a lower light transmittance [49]. The thickness of the chitosan was thinner compared to that of the bio-composite films. A decrease in the transparency of the bio-composite films containing glycerol has also been observed in previous studies [38,50].

## 4. Conclusions

Chitosan bio-composite films with different CNC and glycerol contents were produced using the solution casting technique. Both the tensile strength and the elongation at break of the chitosan film increased in the presence of 2 and 4 wt% CNC, but decreased with 8 wt% CNC. Upon incorporating 4 wt% CNC, both the tensile strength and ductility of the chitosan film were enhanced significantly by 206 and 277%, respectively. The incorporation of 20 wt% glycerol into the bio-composite films increased both their strength and stiffness slightly, but drastically reduced their ductility. The thermal resistance of the chitosan film remained unchanged with the presence of 4 wt% CNC. However, the thermal resistance of the chitosan/CNC composite films was reduced by the introduction of 20 wt% glycerol. The combined use of CNC and glycerol lowered the water absorption of the chitosan film dramatically. Furthermore, the light transmission decreased because of the presence of both CNC and glycerol. Overall, it can be concluded that bio-composite films based on chitosan/CNC/glycerol exhibit great potential for application as an alternative packaging film material.

## Data Availability

The data presented in this study are available on request from the corresponding author.
